# Genome-wide gene expression dataset used to identify potential therapeutic targets in androgenetic alopecia

**DOI:** 10.1016/j.dib.2017.05.001

**Published:** 2017-05-06

**Authors:** R. Dey-Rao, A.A. Sinha

**Affiliations:** Department of Dermatology, Jacobs School of Medicine and Biomedical Sciences, University at Buffalo, 6078 Clinical and Translational Research Center, 875 Ellicott Street, Buffalo, NY 14203, United States

**Keywords:** AGA, Androgenetic alopecia, Bald-scalp, Gene-expression, Microarray, CEL

## Abstract

The microarray dataset attached to this report is related to the research article with the title: “A genomic approach to susceptibility and pathogenesis leads to identifying potential novel therapeutic targets in androgenetic alopecia” (Dey-Rao and Sinha, 2017) [1]. Male-pattern hair loss that is induced by androgens (testosterone) in genetically predisposed individuals is known as androgenetic alopecia (AGA). The raw dataset is being made publicly available to enable critical and/or extended analyses. Our related research paper utilizes the attached raw dataset, for genome-wide gene-expression associated investigations. Combined with several *in silico* bioinformatics-based analyses we were able to delineate five strategic molecular elements as potential novel targets towards future AGA-therapy.

**Specifications Table**

**Table:** 

Subject area	*Dermatology*
Organism/cell	*Homo sapiens*
More specific subject area	*Bald and haired scalp samples from patients diagnosed with androgenetic alopecia.*
How data was acquired	*Affymetrix GeneChip HG-U95Av2*
Data format	*Raw data: CEL files*
Experimental factors	*Comparison of bald and haired scalp (skin) from AGA patients*
Experimental features	*The raw gene expression dataset was preprocessed, and the quality checked by using several QC criteria invoked in Partek Genomic Suite v6.6. The 1-way ANOVA model combined with Fisher׳s Least Significant Difference (LSD) contrast method was used on all preprocessed 12,625 probesets, to compare gene expression in the bald versus haired scalp samples.*
Data source location	*Patients diagnosed with AGA were recruited into the study from the Dermatology Outpatient Clinic of New York Presbyterian Hospital, Cornell University IRB # 0998-398*
Data accessibility	*Data is available with this article*

**Value of the data**

• The raw data files will allow future scientists to re-analyze the data in single- or meta-analyses.
• The raw dataset will allow future scientists to extend statistical analyses.
• The data fills a major gap in knowledge regarding changes that underlie bald scalp-specific manifestations in AGA.
• The data is linked to transcriptional profiling and functional and pathway based analyses to clarify underlying mechanisms of disease.

## 1 Data, materials and methods

The raw data files (CEL files) obtained from microarray analysis, that were used in the analysis and interpretation of our related report are available in Supplementary information.

Sample IDs are:AG5011D
AG5011N
AG7003D
AG7003N
AG7007D
AG7007N

Abbreviations:

AG=Androgenetic alopecia

N: haired scalp

D: bald scalp

RNA was extracted from frozen biopsies of haired and bald scalp samples from 3 AGA-patients. Genome-wide gene expression analysis was performed using the microarray platform: Human Genome U95Av2 GeneChip (Affymetrix, Santa Clara, CA, USA) according to manufacturer׳s protocols. Biotinylated probes were fragmented and hybridized to the microarray chips (containing>12,000 probe sets). Default parameters were used to import the gene expression data to Partek Genomics Suite v6.6 (Partek, St Louis, MO). Raw data preprocessing included log2 transformation, quantile normalization, RMA background subtraction and median polish and was used for probeset summarization to scale mean expression values of all 6 arrays. The preprocessed dataset was subsequently processed to establish differentially expressed genes between bald and haired scalp using the “Gene Expression” tool within Partek Genomic Suite [1].

## Footnotes

Appendix A Supplementary data associated with this article can be found in the online version at doi:10.1016/j.dib.2017.05.001.
